# Sensors and System for Vehicle Navigation

**DOI:** 10.3390/s22051723

**Published:** 2022-02-23

**Authors:** Andrzej Stateczny, Witold Kazimierski, Pawel Burdziakowski

**Affiliations:** 1 Department of Geodesy, Gdansk University of Technology, 80-233 Gdansk, Poland; pawel.burdziakowski@pg.edu.pl; 2 Department of Geoinformatics and Hydrography, Maritime University of Szczecin, 70-500 Szczecin, Poland; w.kazimierski@am.szczecin.pl

## 1. Introduction

In recent years, vehicle navigation, in particular autonomous navigation, has been at the center of several major developments, both in civilian and defense applications. New technologies, such as multisensory data fusion, big data processing, or deep learning, are changing the quality of areas of applications, improving the sensors and systems used. Recently, the influence of artificial intelligence on sensor data processing and understanding has emerged. Radar, LiDAR, visual sensors, sonar systems, and other sensors are mounted onboard smart and flexible platforms and on several types of unmanned vehicles in all types of environments. These technologies focusing on vehicle navigation may encounter many common scientific challenges. Particularly interesting is autonomous navigation for non-GNSS applications, such as underwater and indoor vehicle navigation.

The Special Issue entitled “Sensors and System for Vehicle Navigation” focused on many aspects of vehicle navigation, such as autonomous navigation, multisensor fusion, big data processing for vehicle navigation, sensors related to science/research, algorithms/technical development, analysis tools, synergy with sensors in navigation, data fusion, and artificial intelligence methods for navigation.

Topics for the Special Issue included the following:

- Multisensor data fusion for navigation;
- Sensor-based autonomous navigation;
- Comparative (terrain reference) navigation;
- Aerial vehicle navigation;
- Surface vehicle navigation;
- Underwater vehicle navigation;
- Non-GNSS autonomous vehicle navigation;
- Three-dimensional radar and three-dimensional sonar for vehicle navigation;
- Gravity and geomagnetic sensors for navigation;
- Sensor data processing, data reduction, feature extraction, and image understanding;
- Automatic target and obstacle detection and classification;
- Target tracking and anticollision algorithms and methods;
- Artificial Intelligence for navigation and sensors data processing;
- Big data processing for vehicle navigation;
- Path-planning methods for autonomous vehicle navigation;
- Real-time terrain matching images;
- Close range photogrammetry and commuter vision methods for vehicle navigation;
- Deep learning algorithms for vehicle navigation.

## 2. Overview of Contributions

Breux, Y et al. [1] work has made a significant contribution to the field by introducing a solution for merging the measurements from two perpendicular profiling sonars with different beam widths for underwater cave exploration and mapping. These kinds of environments have very complex geometries that require 3D sensing. Wide-beam sonars are used to cover seen surfaces, but do not provide 3D measurements as the elevation angles are unknown. The method proposed in the paper [1] leverages the narrow-beam sonar measurements to estimate the local karst surface with Gaussian process regression. The estimated surface is then further exploited to infer scaled-beta distributions of elevation angles from a wide-beam sonar. The final results allows one to benefit from the high coverage provided by wide-beam sonars without the drawback of losing 3D information.

The study by Fariña, B. et al. has shed more light on an autonomous wheelchair localization. In the article [2], the authors describe a localization module that includes a combination of various sensors. The sensors used in the module feature variable covariance estimations in order to yield an accurate final localization. In the presented module, all the sensors have a variable covariance estimation that depends on the data quality. A Doppler speed sensor was used to estimate the covariance of the encoder odometric localization. LiDAR was used as a scan matching localization algorithm, comparing the difference between two consecutive scans to obtain the change in position. Matching quality gives the accuracy of the scan matcher localization. The presented structure generates a better position than a traditional static covariance method. 

The study [3] presented by Fukuda, G. et al. focuses on the use of consumer-grade sensors for ship navigation. Generally, global navigation satellite system (GNSS) spoofing poses a significant threat to maritime logistics. Currently, many maritime electronic devices rely on GNSS time, positioning, and speed for safe vessel operation. In the study, authors considered inertial measurement unit (IMU) and Doppler velocity log (DVL) devices in conventional navigation, which are important in the event of GNSS spoofing or outage. A velocity integration method using IMU and DVL in terms of dead-reckoning was investigated. The authors evaluated the performance of a micro electromechanical system (MEMS)-based yaw rate angle with DVL using 60 min of raw data for a 50 m long ship located in and the performance of an IMU-MEMS using three gyroscopes and three accelerometers with DVL. A ship’s gyrocompass was used as a heading reference. The results proved that presented methods achieve less than 1 km horizontal error in 60 min.

A related problem is underlined in the work [4] presented by Gao, B. et al. The study develops and presents a novel robust cubature Kalman filter (CKF) with scaling factor. It establishes a theory of abnormal observations identification using the Mahalanobis distance criterion. Then, a robust factor (scaling factor), which is calculated via the Mahalanobis distance criterion, was introduced into the standard CKF to inflate the observation noise covariance, resulting in a decreased filtering gain in the presence of abnormal observations. The proposed solution is resistant to the influence of abnormal observations on navigation solutions and thus improves the robustness of CKF.

The solution presented by Gioia, C. et al. in [5] introduces a mathematical extension of the traditional Time Difference Of Arrival (TDOA) localization technique, allowing merging TDOA measurement from synchronous and non-synchronous Automatic Identification System (AIS) receivers. The presented technique, derived from satellite applications, was tested in a simulated scenario where the position of a moving target was estimated using different configurations of the receiver’s network. The proposed approach can be adopted to estimate the position of any radiofrequency transmitter by employing a suitable number of non-synchronous receivers.

Hyun, E. et al. in their work [6] introduced three novel features, referred to as the scattering point count, scattering point difference, and magnitude difference rate features, based on the characteristics of the Doppler spectrum in two successive frames for frequency-modulated continuous wave (FMCW) radar sensor. Using 24 GHz FMCW radar front-end module and a real-time data acquisition module, thanks to the presented solution, the authors reached the average performance exceeded 99% and 96% for the walking human and the moving vehicle, respectively.

Jamil, S. et al. [7] provided a novel framework consisting of a hybrid handcrafted and deep feature to detect and localize malicious drones from their sound and image information. The authors applied various kernels of the support vector machine (SVM) method to classify the drone features. Using a big dataset, including sounds, occluded images of birds, airplanes, and thunderstorms, with variations in resolution and illumination, the authors proved the improved performance of the proposed scheme in comparison to other related methods.

Karbowska-Chilinska, J. et al. [8] took on one of the most important tasks in vehicle navigation: avoiding collision. In the case of maritime environments, the biggest challenges are accidents and excessive fuel consumption. In this paper, the strategy of anti-collision, shortest trajectory planning based on the beam search method, is proposed in order to improve the safety of navigation. The proposed algorithm generates many safe trajectories for the present ship and chooses the best in terms of length. The collision risk is detected when the closest point of approach (CPA) of the present ship is crossed by a target ship’s planned trajectory. The resultant trajectory is determined on the basis of the navigation data from the perspective of the given ship and the present ship.

This paper of Kolar, P. et al. [9] focuses on data fusion, which is fundamental to one of the most important modules in any autonomous system: perception. Autonomous systems can play a vital role in assisting humans in a variety of problem areas. This could potentially be in a wide range of applications, such as driverless cars, humanoid robots, assistive systems, domestic systems, military systems, and manipulator systems. Presently, the world is seeing an increase in technologies that can enable this, even in our daily lives. The presented survey focuses on the vehicle navigation of an autonomous vehicle. The article reviews a various types of sensors, as well as their data, and the call for fusion of the data to output the best data for the task at hand, which in this case is autonomous navigation. The authors review the past and present research using Light Imaging Detection and Ranging (LiDAR) and imaging systems, such as a camera, which are laser- and vision-based sensors, respectively. Then, the revision of fusion is given. The autonomous systems use sensor data for several tasks, such as object detection, obstacle avoidance, mapping, localization, etc. As a conclusion, a comparison of the different types of data fusion and their pros and cons is provided.

The paper of Lewicka, O. et al. [10] undertakes the problem of the integration of hydrographic data. The aim of the review is to present selected fusion methods applying the data derived from Global Navigation Satellite System (GNSS), Real Time Kinematic (RTK) measurements, hydrographic surveys, a photogrammetric pass using unmanned vehicles, and Terrestrial Laser Scanning (TLS) and compare their accuracy. Each of the hydroacoustic and optoelectronic systems used in hydrography is characterized by a different spatial reference system and the method for technical implementation of the measurement. Therefore, the integration of these data requires that problems in selected fields of electronics, geodesy, and physics (acoustics and optics) be solved. This paper analyzes selected methods of data integration, which additionally presents the process of data acquisition and processing. The method review contributes significantly to the development of the data integration model. The analyzed examples show that there is no single data fusion scheme. This is due to the different specifications of the devices used, research aims, and types of waterbodies. In all schemes, the data fusion was multi-stage and required the use of commercial software, such as ArcGIS, CloudCompare, and VDatum. The integration of hydroacoustic and optoelectronic data is a new issue that requires detailed study. An alternative to complex methods of spatial data integration are machine learning methods, which, using artificial intelligence, automate the process of creating models. The model is built on the training set, which are model patterns. One of the machine learning methods is Artificial Neural Networks (ANNs), which are used to transform coordinates with a small number of references or to create DTMs. Machine learning methods will help to improve the accuracy of integrated data, assuming that the training set and the analyzed data come from the same devices and systems.

López, J. et al. [11] provide a new approach to implement autonomous driving behaviors using a discrete event model framework. The article deals with the typical problems of the executive layer in the autonomous decision-making system for cars. Most autonomous car control frameworks are based on a middleware layer with several independent modules that are connected by an inter-process communication mechanism. These modules implement basic actions and report events about their state by subscribing and publishing messages. In their study, the authors propose an executive module that coordinates the activity of these modules. The whole system was applied to an autonomous car designed for elderly or disabled people. The solution proposed in the paper can be implemented in any control framework that includes a functional layer with a set of components that provide access to sensor data, send commands to actuators, and execute different basic functionalities.

In the paper of Moreno, F.-A. et al. [12], a practical solution is given to the navigation of autonomous robots through narrow spaces that automatically foster the robot to traverse such difficult areas following a straight path. The authors propose an automatic procedure for domestic robotics. The procedure includes passing through narrow areas, detecting problematic areas, and generating a set of auxiliary navigation waypoints from which more suitable trajectories can be generated by the robot planner. The method offers the identification, without any human intervention, of cumbersome zones in the robot’s working area during an initial inspection stage, and the automatic and on-the-fly generation of a pair of auxiliary navigation waypoints for each cumbersome zone, which modify the robot trajectory and ensure proper navigation through such zones. 

Moussa, M. et al. [13] propose a new high-resolution direction of arrival (DOA) estimation method of jammers. The paper studies high-resolution DOA estimations for interference detection using the uniform linear array (ULA) and uniform circular array (UCA) configurations. The authors contribute to the filed by the use of nonlinear signal modeling techniques for GPS jamming detection with ULAs and UCAs. Thanks to this method, the anti-jamming process can be significantly improved by limiting the erroneous spatial attenuation of GPS signals arriving from an angle close to the jammer. The proposed method was more accurate at detecting the amplitudes of the single and multiple jammers than the one that was referenced.

Naus, K. et al. [14] presented the results of their research on the assessment of the accuracy of angular position measurements relative to the sea horizon using a camera mounted on an unmanned bathymetric surveying vehicle of the Unmanned Surface Vehicle (USV) or Unmanned Aerial Vehicle (UAV) type. Their proposed method is based on observing the horizon line slope in the camera image to determine the spatial orientation angles. A mathematical description of the meters characterizing a resolution and a mean error of measurements, made on the base of the horizon line image, was provided. The method was tested in a real environment, based on an UAV flight.

Nguyen, H.T. et al. [15] present their research on the detection of objects in sonar images and 3D LiDAR data with the use of deep learning algorithms. The detection of multiple objects is crucial in image processing. It has been investigated extensively owing to its potential wide application in numerous fields, such as computer vision, machine inspection, manufacturing industry, and self-driving cars. In this study, several deep learning and clustering algorithms were investigated, including underwater sonar image data for multiple submerged human bodies and object detection, and three-dimensional (3D) LiDAR data for multiple object classification and segmentation in an urban structure to be applied in autonomous driving. The results of the study may be applicable for detecting multiple objects in both the underwater and terrestrial environments to study underwater sciences and autonomous driving systems, respectively.

Rubio-Sierra, C. et al. [16] presented a path planning solution that enables the autonomous exploration of underground mines using aerial robots (typically multicopters). The presented path planner was defined as a simple and highly computationally efficient algorithm that relied only on a laser-based sensor with simultaneous localization and mapping capabilities. Their solution allows the exploration of a set of single-level mining tunnels. The performance of the proposed solution was tested in different test cases using a hardware-in-the-loop simulator developed for this purpose. The solution is very fast and works in real-time using static memory-equipped algorithm. Their solution provides a viable solution for the autonomous exploration of underground mines.

Specht, M. [17] proposes a method that allows any navigational positioning system to be evaluated for its compliance (or non-compliance) with the minimum accuracy requirements specified for hydrographic surveying. The discussed method explicitly evaluates whether a given positioning system meets the accuracy requirements specified for a particular IHO (International Hydrographic Organization) assignment. The model was verified considering both past and present test results (station and dynamic) from tests on the following systems: DGPS (Differential Global Positioning System), EGNOS (European Geostationary Navigation Overlay Service), and multi-GNSS (GPS/GLONASS/BDS/Galileo) receivers. The tests confirmed that the DGPS system meets the requirements for all IHO orders and showed that the EGNOS system can currently be used for measurements in orders 1a, 1b, and 2. On the other hand, multi-GNSS receivers meet the requirements for order 2, and some of them also for orders 1a and 1b.

The primary means of electronic positioning currently used on most modern commercial vessels are shipboard GPS (Global Positioning System) or DGPS (Differential GPS) receivers and IALA (International Association of Lighthouse Authorities) radio beacon receivers. More advanced GNSS (Global Navigation Satellite System) receivers, capable of processing signals from GPS, Russian GLONASS, Chinese Beidou, European Galileo, Indian IRNSS, Japanese QZSS, and satellite-based augmentation systems (SBAS) are still relatively rare at sea. However, it is expected that such combined or multisystem receivers, integrated with gyroscopic, inertial, radar, laser, and optical sensors, will soon become increasingly common in maritime transportation and indispensable aboard maritime autonomous surface ships (MASS). To be prepared for the failure of any of the position sensors, their state-of-the-art integrity monitoring system must be developed and standardized, taking into account the specifics of MASS and e-navigation safety. Zalewski, P. [18] discusses and presents issues regarding existing requirements, performance standards, and future concepts for the integrity monitoring of marine position sensors.

A bionic autonomous positioning mechanism integrating INS with bioinspired polarization compass is proposed by Zhang, Q. et al. [19] to solve the problem of positioning error accumulation of an inertial navigation system (INS). The hardware for building the bioinspired positioning system and the integration model are also presented. Regarding the technical issue of the accuracy and environmental adaptability of the integrated positioning system, a method for calculating solar altitude based on the degree of polarization and polarization direction (E-vector) is presented. Furthermore, to compensate for the latitude and longitude errors of the INS, a bio-inspired positioning system model combining a polarization compass and INS is established. Finally, the positioning performance of the proposed bioinspired positioning system model is verified through outdoor experiments. The results indicate that the proposed system can compensate the INS position errors with satisfactory performance.

## 3. Conclusions

The Special Issue entitled “Sensors and System for Vehicle Navigation” comprised 19 articles on many topics related to the navigational aspects of sensors and systems for vehicle navigation also focused on autonomous navigation fusion. In this paper, brief introductions to the published articles are provided.

It can be said that in-vehicle navigation, and autonomous navigation in particular, is still an important and popular topic, and much work will continue to be undertaken in this area around the world. New techniques and methods for analyzing and extracting information from sensors and systems have been proposed and verified. Some of them will provoke further research, and some are already mature and can be considered for industrial implementation and development.
